# Photocatalytic degradation of organic dye and tetracycline by ternary Ag_2_O/AgBr–CeO_2_ photocatalyst under visible-light irradiation

**DOI:** 10.1038/s41598-020-76997-0

**Published:** 2021-01-08

**Authors:** Fu Su, Pengpeng Li, Jianshu Huang, Meijuan Gu, Zhiying Liu, Yanhua Xu

**Affiliations:** 1 Nanjing Tech University, Nanjing, 211800 People’s Republic of China; 2 School of Chemistry and Materials Science, Nanjing Normal University, Nanjing, 210023 People’s Republic of China

**Keywords:** Photocatalysis, Catalytic mechanisms, Catalyst synthesis

## Abstract

In this work, CeO_2_ nanosheets decorated with Ag_2_O and AgBr are successfully fabricated via a simple sediment-precipitation method. The as-prepared ternary Ag_2_O/AgBr–CeO_2_ composite with double Z-scheme construction was analyzed by various analytical techniques. Ag nanoparticles (NPs) used as the electron medium could reduce the recombination of photoelectrons and holes, thus leading to the improvement of photocatalytic performance of these catalysts. Due to the unique structure and composite advantages, the optimal Ag_2_O/AgBr–CeO_2_ photocatalysts exhibit the superior tetracycline (TC) degradation efficiency of 93.23% and favorable stability with near-initial capacity under visible light irradiation. This ternary Z-scheme structure materials will be the well-promising photocatalysts or the purification of antibiotic wastewater.

## Introduction

With the unreasonable use of antibiotics in human production and life, antibiotics which come from the wastewater of medical treatment^1^, agriculture^2^, animal husbandry^3^ and industry^4^ have become an important pollutant that contaminates the environment, especially the antibiotic wastewater represented by tetracycline (TC). Recently, a variety of methods such as physical absorption, biodegradation, et al. have been developed to eliminate pollution of TC^5,6^. Among the above methods, photocatalytic degradation is one of the most potential because of its low mammalian toxicity and high-efficiency. Nonetheless, the great mass of semiconductors such as TiO_2_, ZnO et al. can only degrade the contaminants under the UV light, which reduces the utilization effect of visible light^7^. Therefore, the quest for an ideal visible light absorption photocatalyst has become the focus on attention.

Cerium oxide (CeO_2_) is a potential photocatalyst because of its outstanding catalytic activities, lower production costs, and excellent optical and chemical properties^8^. However, its widespread application under visible light irradiation is hindered owing to its over quick recombination of photoelectron hole pairs and the narrow range of light responses^9^. Recent years, a variety of coping methods such as morphological control^9^, doping other elements^10^, heterojunctions engineering^11^ have been adopted to remedy the shortcomings, thus improving the catalytic activity of CeO_2_. Among them, constructing heterojunctions can effectively improve catalytic performance due to the enhanced light absorption ability and effective charge transfer rate. And the increasing number of heterojunctions such as C_3_N_4_/CeO_2_^12^, BiOI/CeO_2_^13^, AgI/CeO_2_^14^ and Co_3_O_4_/CeO_2_^15^, are reported to apply for photocatalytic degradation of TC. Although the photocatalytic activity of binary composite has a great improvement, it is still some distance from practical application.

The Z-scheme photocatalytic system originates from the light reaction stage of plant photosynthesis in nature^16^. The Z-scheme photocatalytic system shows strong reducibility of PS I (Photocatalytic System I) and strong oxidization of PS II. The difference is that oxidation reaction and reduction reaction of traditional heterojunctions occur in the valence band and conduction band of PS I and PS II, respectively, although they can effectively improve the photoelectron-hole separation. However, due to the relationship between PS II and PS I position, although the range of light response is widened, the ability of REDOX will be weakened. Compared with the traditional heterojunction, Z-scheme photocatalytic materials can not only ensure the expansion of the optical response range, but also improve the oxidation and reduction capacity of PS I and PS II. The Z-scheme photocatalytic materials will have an excellent application prospect.

In recent years, more and more researchers have paid attention to the preparation of ternary composites. In the whole ternary catalytic reaction process, photo-generated carriers can be transferred in multiple steps through the induction mechanism to achieve the purpose of electron and hole separation, thereby achieving photocatalytic activity beyond the binary catalytic system^17,18^. And an increasing number of ternary photocatalytic materials have been reported, such as Fe_3_O_4_/Bi_2_S_3_/BiOBr^19^, Ag_3_PO_4_/TiO_2_/Fe_3_O_4_^20^, Bi_2_WO_6_/Ag_2_S/ZnS^21^, and V_2_O_5_/BiVO_4_/TiO_2_^22^. In spite of some ternary composites reported, there are few reports about cerium dioxide ternary materials. Therefore, it is expected to design a preeminent three-way CeO_2_-based photocatalyst with favorable catalytic properties.

It’s necessary to select suitable doping materials which could directly affect the efficiency of whole ternary composites, to design the ternary CeO_2_-based photocatalysts. Ag_2_O has excellent visible light absorption properties, while AgBr has excellent photocatalytic activity^23,24^. Compared to the existing materials, the two may be the better choice. However, pure Ag_2_O and AgBr also have problems such as lower photo-generated carrier yield, unstable photocatalytic activity and high carrier recombination rate. To address these shortcomings, researchers often use them as co-catalysts to improve their stability. For example, Wen et al. fabricated Ag_2_O–CeO_2_ photocatalyst to degrade enrofloxacin effectively by Ag_2_O nanoparticles embellishing CeO_2_ spindles^25^. Huang et al. synthesized flower-like AgBr/Bi_2_WO_6_, which degraded 87.5% TC solution within 60 min under visible light irradiation^6^. Thus, we attempt to modify cerium dioxide with silver oxide and silver bromide to construct Ag_2_O/AgBr–CeO_2_ ternary composites.

## Results and discussion

### Physical and chemical properties of samples

**Figure 1:**
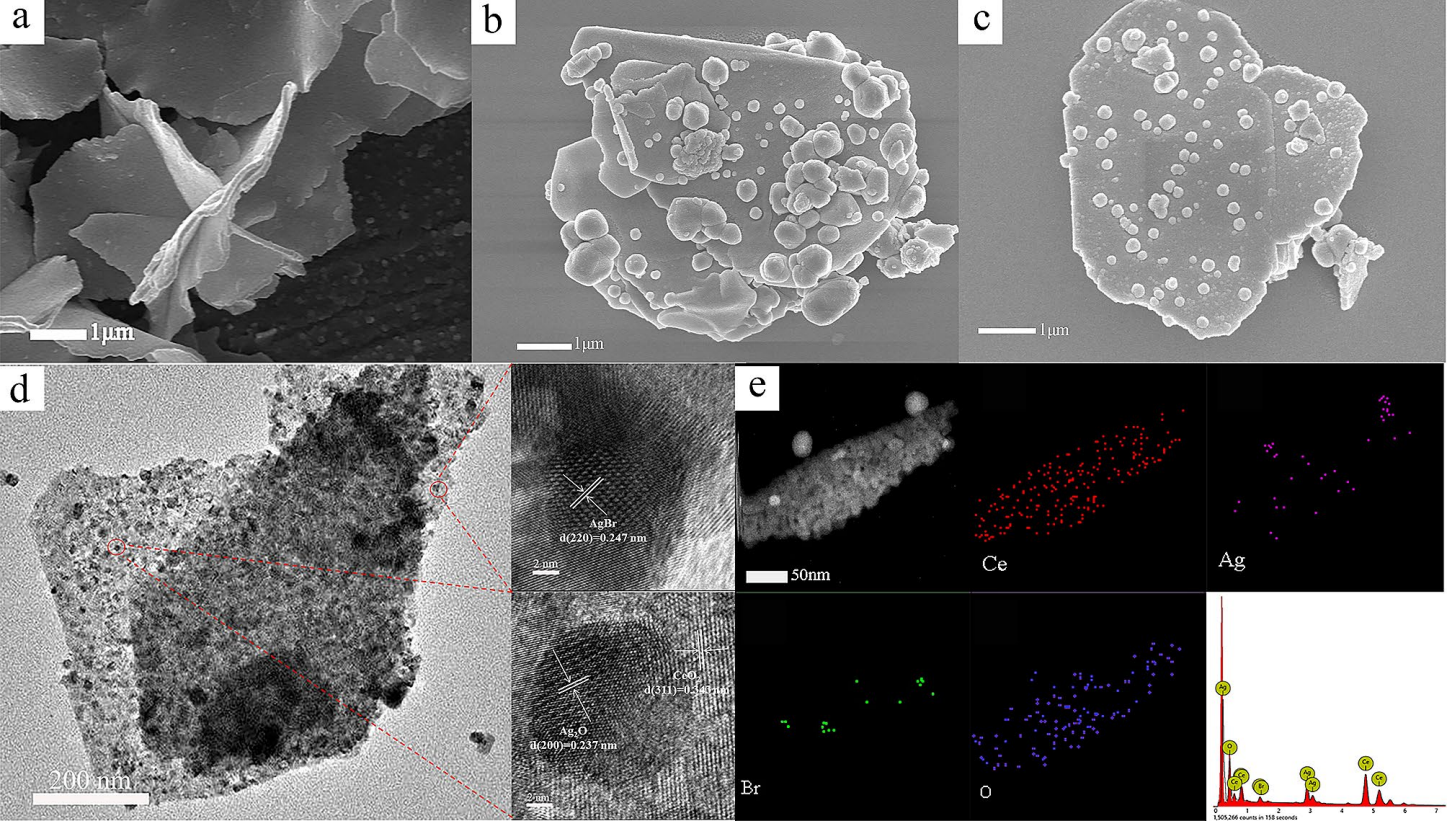
SEM images of (**a**) CeO_2_, (**b**) Ag_2_O–CeO_2_ and (**c**) ACA-2; (**d**) TEM and HRTEM images of ACA-2; (**e**) EDX mapping of ACA-2.

The SEM images of CeO_2_, Ag_2_O, and ACA-2 were shown in Fig. 1. In Fig. 1a, the length and the thickness of CeO_2_ nanosheets are about 1–5 μm and 50–100 nm, respectively. The size of Ag_2_O NPs is about 206 nm (Supplementary Fig. S1), but most of Ag_2_O NPs were agglomerated. In Fig. 1b, the particle size of Ag_2_O was 100–400 nm and relatively inhomogeneous. In Fig. 1c, after decoration with Ag_2_O and AgBr NPs, it was clearly to be seen that Ag_2_O spreads over the surface of CeO_2_ with the lower agglomeration of Ag_2_O. Ag_2_O and AgBr NPs stick well to the surface of CeO_2_ nanosheets. The grain sizes of Ag_2_O and AgBr NPs were more uniform. The main reason is that the corrosion of hydrobromic acid makes silver oxide release Ag. Subsequently, the Ag NPs are evenly distributed on the surface of CeO_2_ to form homogeneous AgBr NPs^17^. Therefore, the above result confirmed that the preparation of the three-way Ag_2_O/AgBr–CeO_2_ catalyst is successful.

The TEM and HRTEM were applied to further analyse the structure of the ACA-2. In the picture of Fig. 1d, a large number of Ag_2_O and AgBr NPs with an average diameter below 30 nm were grown along the surface of CeO_2_ microsheets. The (311) crystal plane of CeO_2_ was 0.343 nm^26^. The observed lattice spacing of 0.247 and 0.237 nm in HRTEM image are separately consistent with the (220) crystal plane of AgBr^27^ and (200) crystal plane of Ag_2_O^28^, respectively. Meanwhile, Selected Area Electron Diffraction (SAED) Patterns could also confirm the theory (Supplementary Fig. S2). The photos of TEM–EDX mapping are exhibited in Fig. 1e. TEM–EDX mapping was used to analyze each element of the sample. As shown in Fig. 1e, the Ce, O and Ag elements evenly distributed on the sample surface. The Br element is distributed in small amounts on the surface, thus proving that AgBr exists on the surface of CeO_2_. Meanwhile, EDX Spectrum of ACA-2 further proves the presence of Ce, O, Ag, and Br elements within the samples. As shown in Supplementary Table S1, the weight percent of Ce and O is 54.84% and 24.73%. The actual amount of element Ag and Br is 16.84% and 3.6%, which suggests that Ag_2_O and AgBr are loaded on the CeO_2_ successfully. Meanwhile, EDX Spectrum of ACA-2 further proves the presence of Ce, O, Ag, and Br within the samples.

**Figure 2:**
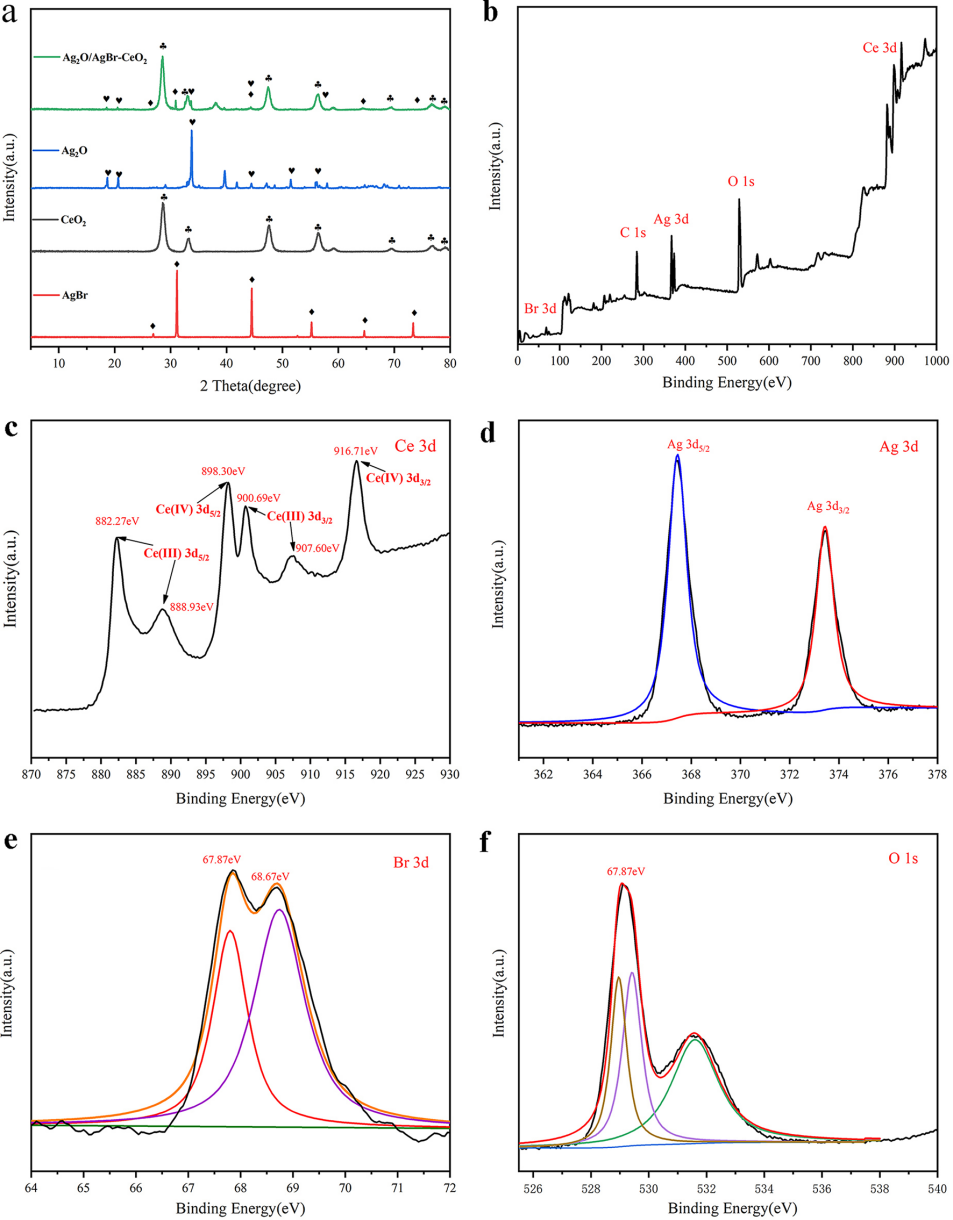
(**a**) XRD patterns of CeO_2_, Ag_2_O, AgBr and ACA-2 composites and (**b**–**f**) XPS spectrum of ACA-2.

The XRD patterns of pure Ag_2_O, AgBr, CeO_2_, and ACA-2 samples are described in Fig. 2a. It is evident that the main diffraction peaks of pure CeO_2_ samples at 2*θ* values of 28.48°, 33.02°, 47.42°, 56.29°, 59.05°, 69.33°, 76.66°, and 79.10° correspond to the (111), (200), (220), (311), (222), (400), (331) and (420) planes of the cubic phase CeO_2_ crystal (JCPDS: 34-0394)^27^. Moreover, other crystal phases cannot be caught in the XRD pattern, indicating that the crystallinity of synthesized CeO_2_ is favorable. The sample of pure Ag_2_O shows the typical diffraction peaks at 2*θ* of 32.76°, 38.10°, 54.74°, 65.41°, and 68.69°, which corresponds to the (110), (111), (200), (220), (331) and (222) planes (JCPDS: 41-1104)^25^. The diffraction peak of AgBr standard cards (JCPDS: 06-0438)^29^ correspond to XRD patterns of the pure AgBr. Respecting the ACA-2 composites, the typical diffraction peaks of CeO_2_, AgBr, and Ag_2_O can be clearly observed. According to Supplementary Fig. S3, it could also be clearly observed that the peak of AgBr of each material become more obvious with the increase of HBr added amount. The unobvious peaks of Ag_2_O due to the shading effect of the close peaks of CeO_2_^25^. According to the above analysis, the crystal phase of Ag_2_O/AgBr–CeO_2_ was not affected by loading the Ag_2_O and AgBr.

X-ray photoelectron spectroscopy (XPS) tests were carried out to determine the elemental composition and the chemical state of ACA-2. The XPS survey spectrum in Fig. 2b shows that the product contains Ce, O, Ag, and Br elements. There are four peaks of Ce (III) spectra at 882.27, 888.93, 900.69, and 907.60 eV. And two peaks of Ce (IV) at 898.30 and 916.71 eV in Fig. 2c, which is in keeping with previous reports^30^. The positions of Ag 3d_5/2_ and Ag 3d_3/2_ peaks are at 367.44 and 373.45 eV (Fig. 2d), respectively, illustrating the monovalent chemical valence of Ag^5^. The peaks of Br 3d can be assigned to Br 3d_5/2_ (67.87 eV) and Br 3d_3/2_ (68.67 eV) in Fig. 2e^31^. As presented in Fig. 2f, it is observed that O 1 s has two peaks (Fig. 2f) of Ce–O and Ag–O bonds. Besides, the other peak is related to absorbed oxygen and H_2_O^27^. Therefore, the consequence of XPS analysis indicates that Ag_2_O and AgBr connect with CeO_2_ via chemically bound interface rather than the physical contact.

**Figure 3:**
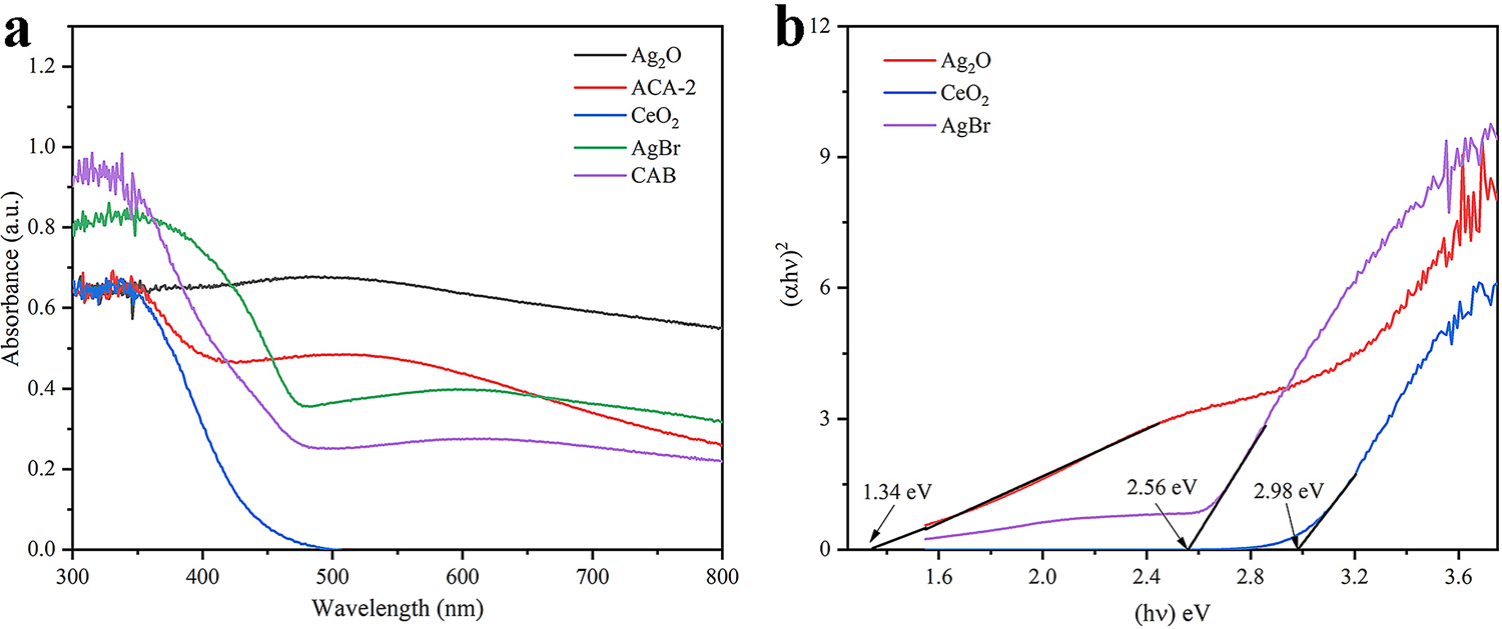
(**a**) UV–Vis DRS of CeO_2_, Ag_2_O, AgBr, AOC and ACA-2; (**b**) bandgap diagram of Ag_2_O, CeO_2_ and AgBr.

UV–Vis spectra were illustrated in Fig. 3a. CeO_2_ and AgBr exhibited visible light absorption with absorption band edges at 436 and 519 nm, respectively^32,33^. Meanwhile, pure Ag_2_O revealed an evident absorption in completely visible light scope, which corresponds with the previous reports^34^^.^ As shown in Fig. 3b, the band-gap (E_g_) could be obtained by Kubelka–Munk function: *α*h*v* = A (h*v*—*E*_*g*_)^n/2^, where *E*_*g*_, *α,* A, h, *ν* correspond to energy gap, absorption coefficient, a constant, Planck’s constant and light frequency. CeO_2_, Ag_2_O and AgBr were indirect bandgap, and four was the value of n^35^. The *E*_*g*_ values of CeO_2_, AgBr and Ag_2_O were about 2.98, 2.56 and 1.34 eV, respectively.

According to the empirical formulas *E*_*CB*_ =*X* − *E*_*C*_ − 0.5*E*_*g*_ and *E*_*VB*_ = *E*_*CB*_ + *E*_*g*_, where *X, E*_C_ and *E*_g_ represented the electronegativity of crystalline semiconductors, the energy of free electrons on the hydrogen scale (~ 4.50 eV NHE) and the energy gap of semiconductors^36,37^. The *E*_*CB*_ of CeO_2_, Ag_2_O and AgBr were calculated to be − 0.39, 0.13 and 0.02 eV. Then their *E*_*VB*_ was corresponded to 2.59, 1.47, and 2.58 eV.

### Photocatalyst behaviors

**Figure 4:**
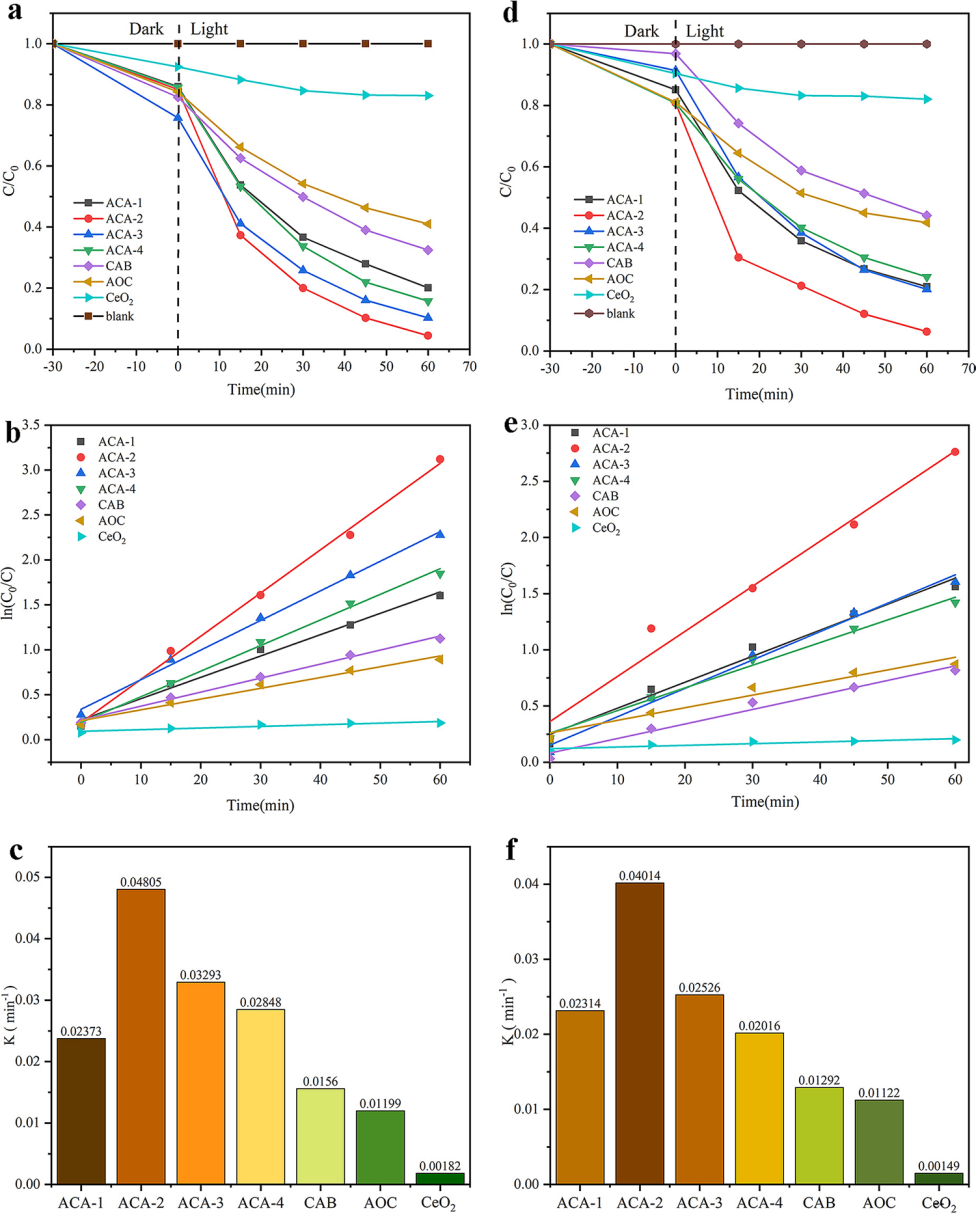
(**a–c**) Photocatalytic degradation RhB curves and Kinetic curves of RhB degradation; (**d–f**) Photocatalytic degradation TC curves and Kinetic curves of TC degradation.

The photocatalytic performances of the samples over RhB were studied under visible light. The details of the dark reaction experiments were shown in Supplementary Fig. S4. From Fig. 4a, the degradation of RhB did not proceed in the blank experiment, which illustrates RhB can’t be degraded under visible irradiation. The degradation rates of RhB for AOC and CAB were 58.48% and 67.55% within 60 min. And degradation rate of pure CeO_2_ was only 16.99%. For Ag_2_O/AgBr–CeO_2_ composites, the ACA-2 sample showed the best photocatalytic activity, which can remove 95.59% RhB. It could be discovered that the quantity of AgBr affected their ability of degradation. The degradation rate was increased because the purity of AgBr was from 7.92 to 15.37 wt%. When the content of AgBr was exceeded 15.37 wt%, its shading effect had an impact on the photocatalytic activity of composites. As shown from the photocatalytic degradation reaction kinetics of RhB in Fig. 4b,c, the kinetic constant of ACA-2 (0.04805 min^−1^) based on the muri-Hinshelwood model was higher than other samples^38^. However, the kinetic constant of CeO_2_ was only 0.00182 min^−1^. According to the analysis in Fig. 4b, the appropriate addition of AgBr could enhance the catalytic property.

Moreover, TC was selected as a typical antibiotic pollutant, and the obtained samples were degraded to eliminate the influence of dye self-sensitization. The details of the dark reaction experiments were shown in Supplementary Fig. S5. As shown in Fig. 4d, only 17.95% and 55.84% of TC could be removed by pure CeO_2_ and AOC after 60 min of visible light irradiation, respectively. As expected, when AgBr nanoparticle put into composites, the photocatalytic activity of ACA-2 (93.68%) was more outstanding than other materials. The catalyst exhibited evidently degradation effect was analogous to its catalytic behavior of removal RhB. Meanwhile, the degradation effect kinetic of TC for all products were studied in Fig. 4e,f. Kinetic constants of ACA-1, ACA-2, ACA-3, ACA-4, CAB and AOC were to 0.02314, 0.04014, 0.02526, 0.02016, 0.01292 and 0.01122 min^−1^, while CeO_2_ was only 0.00149 min^−1^. The information could prove that the photocatalytic activity of CeO_2_ could be improved by Ag_2_O and AgBr modification.

**Figure 5:**
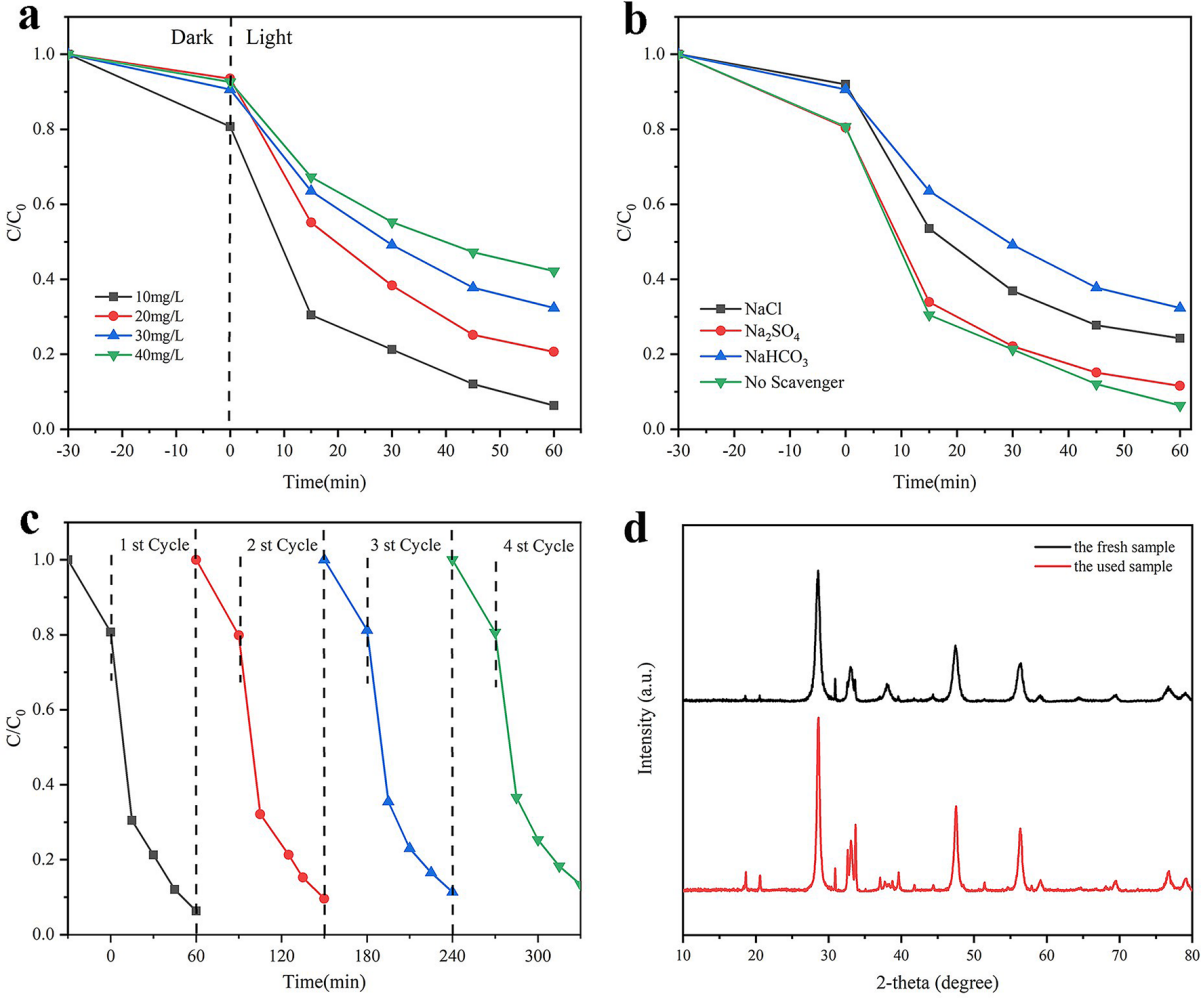
(**a**) The influence of the initial TC concentration; (**b**) coexistence ions on the photocatalytic activity of ACA-2; (**c**) Cycling test for the photocatalytic degradation of RhB in presence of ACA-2; (**d**) XRD patterns of the fresh and used samples.

As a matter of fact, the concentration of the degradant had an outstanding impact on the photocatalytic activity of Ag_2_O/AgBr–CeO_2_. In Fig. 5a, different concentration of TC was removed by ACA-2. It was obvious that the degradation effect of 10 mg/L TC was 93.84% which was higher than the TC solutions of 20, 30 and 40 mg/L. The main reasons for the attenuation of degradation activity could be the following influences: (1) high beginning concentrations of TC may increase the path length of photons entering the reaction and reduce the number of photons on the surface of catalysis^36^. (2) As the concentration of pollutants increases, the number of intermediates produced in the reaction increases, which led to the competition with TC^39^. Meanwhile, for TOC degradation, ACA-2 also shows the best removal rate (Supplementary Fig. S8).

It is well known that there are many kinds of anions in practical wastewater, which may affect the degradation efficiency of polluted solution by photocatalysts. Therefore, we studied the effects of various anions (SO_4_^2−^, Cl^−^, and HCO_3_^−^) on photocatalytic degradation of TC solution using ACA-2. As shown in Fig. 5b, the degradation rate of TC solution with Na_2_SO_4_ decreased slightly^40^. However, when NaCl appended to the solution, the removal efficiency of TC was reduced to some extent, which might put down to competitive adsorption of Cl^-^ with other substances^38^. The addition of NaHCO_3_ has a significant effect on the degradation of TC because HCO_3_^-^ has the function of a free radical scavenger, which can consume some active free radical^27^. Therefore, the reduction of radicals decreased the photocatalytic degradation of TC by ACA-2.

As a matter of fact, the excellent recycle property and stability of photocatalysts can effectively cut the waste water treatment cost and avert secondary pollution. The stability of ACA-2 was conducted by 4 recycling experiments in Fig. 5c. Specifically, even in the fourth cycle, Ag_2_O/AgBr–CeO_2_ composites could degrade 86.64% TC. According to the SEM and TEM images of used ACA-2 (Supplementary Fig. S6), the micro-morphology of ACA-2 has not been destroyed after multiple reactions. From Fig. 5d, the XRD pattern of the sample after degradation reactions basically corresponded to the fresh sample. The consequence indicated that the stability of Ag_2_O/AgBr–CeO_2_ was excellent.

**Figure 6:**
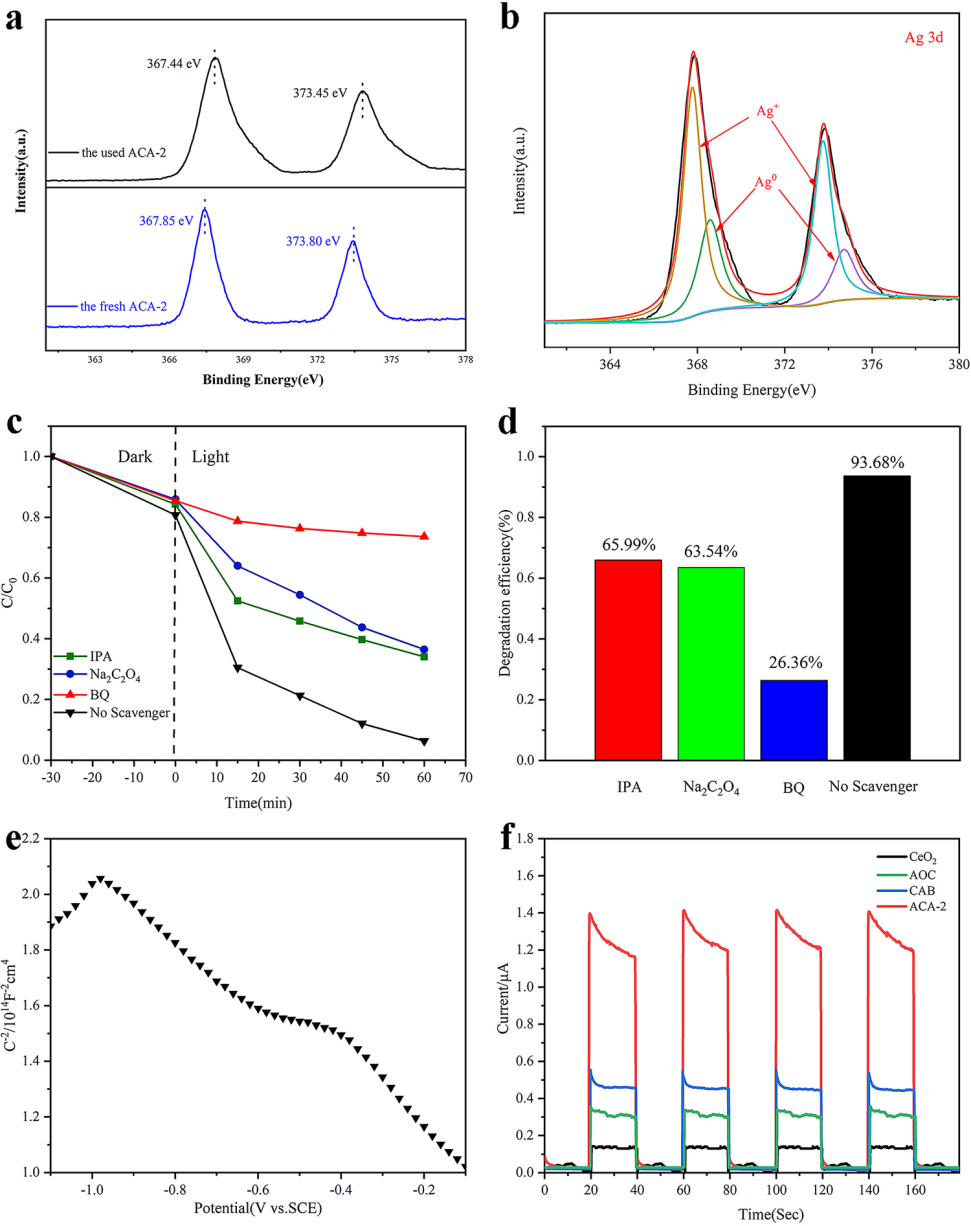
(**a**) Ag XPS spectrum of the ACA-2 before and after photoreaction; (**b**) Ag XPS spectrum of ACA-2 after photoreaction; (**c**) effects of reactive species scavengers on the RhB degradation performance of ACA-2 and (**d**) degradation rate; (**e**) Mott–Schottky curve of ACA-2; (**f**) Transient photocurrent response for CeO_2_, AOC, CAB and ACA-2 composites under visible light irradiation.

In order to investigate the sample changes before and after the reaction, XPS analysis was performed on the samples after the photocatalytic reaction. According to Fig. 6a,b, there are two peaks of Ag in two valence states, which shows that silver nanoparticles appear on the surface of the material. And Ag NPs could reduce photo corrosion and inhibit the photolysis of AgBr and Ag_2_O. In addition, Ag NPs can serve as an electronic medium in the catalytic process.

### Photocatalytic mechanism

To research the photocatalytic mechanism of Ag_2_O/AgBr–CeO_2_ composites, free radical capture experiments were carried out. Three different trapping agents of isopropyl alcohol (IPA, **·**OH scavenger), sodium oxalate (Na_2_C_2_O_4_, **·**h^+^ scavenger) and benzoquinone (BQ, **·**O_2_^−^ scavenger)^41,42^ were added to the solution of TC before photodegradation. As presented in Fig. 6c,d, the degradation of TC hardly reduced after the addition of BQ, explaining that the **·**O_2_^−^ played an important role in reaction systems. Meanwhile, when Na_2_C_2_O_4_ and IPA were added into the TC solution, the degradation rate of TC was 63.54% and 65.99% respectively, less than the removal of no scavenger. Therefore, holes and **·**OH also played a pivotal role in the catalysis system. It can be deduced that the h^+^, **·**OH and **·**O_2_^−^ were the main active radicals affecting the elimination of TC solutions.

As shown form the Mott-Schottky curve of ACA-2 (Fig. 6e), the ternary Ag_2_O/AgBr–CeO_2_ catalyst is composed of p-type semiconductor Ag_2_O and n-type semiconductor CeO_2_ and AgBr. The p–n heterojunction is effectively constructed in the composite catalyst, which can effectively separate the electron-hole^43^. On the side, photocurrent density could be an efficient method of evaluating the transfer properties of the photogenerated electrons as well. And the higher the photocurrent response the higher separation efficiency. From Fig. 6f, these curves of photocurrent density of CeO_2_, AOC, CAB and ACA-2 composites under illuminating visible light were measured. These composites and CeO_2_ showed the stable photocurrents under visible light irradiation respectively generating electrons and holes^44^. And the result of EIS spectra of ACA-2 (Supplementary Fig. S7a) was consistent with that of photocurrent curves. It was obvious that ACA-2 displayed the best excellent photocurrent density curve that illustrated the better separation efficiency of photogenerated electron–hole pairs than others^45^. Generally speaking, the lower the fluorescence intensity of PL spectrum, the better the electron hole separation efficiency of catalyst. According to Supplementary Fig. S7b, ACA-2 has the lowest fluorescence intensity which indicates that it has better catalytic capacity.

**Figure 7:**
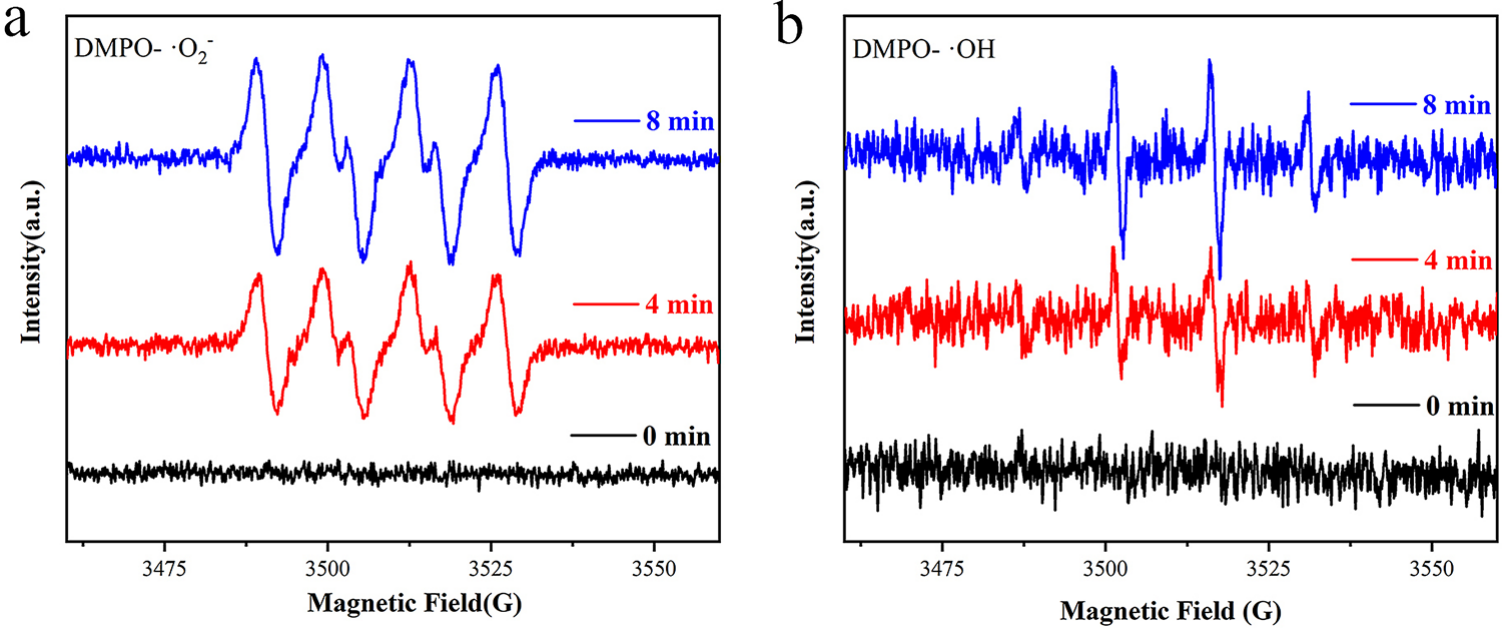
EPR spectra of ACA-2 for detecting (**a**) DMPO **·**O_2_^−^ and (**b**) DMPO **·**OH under the visible light irradiation.

In order to verify the activity of free radicals, the samples were tested by Electron Paramagnetic Resonance (EPR)^46^. It can be seen from Fig. 7a that there is no EPR signal under dark conditions. However, the characteristic signal of **·**O_2_^−^ appears under visible light irradiation, and the signal intensity gradually increases with the increase of the irradiation time (1–8 min), indicating that **·**O_2_^−^ are generated in the reaction system and participate in the photocatalytic degradation reaction. According to Fig. 7b, the characteristic signal of ·OH is also no signal under dark conditions, and gradually increases with the prolongation of the light time (1–8 min), which indicates that **·**OH participate in the photocatalyst reaction. It is consistent with the results of active species trapping experiments.

**Figure 8:**
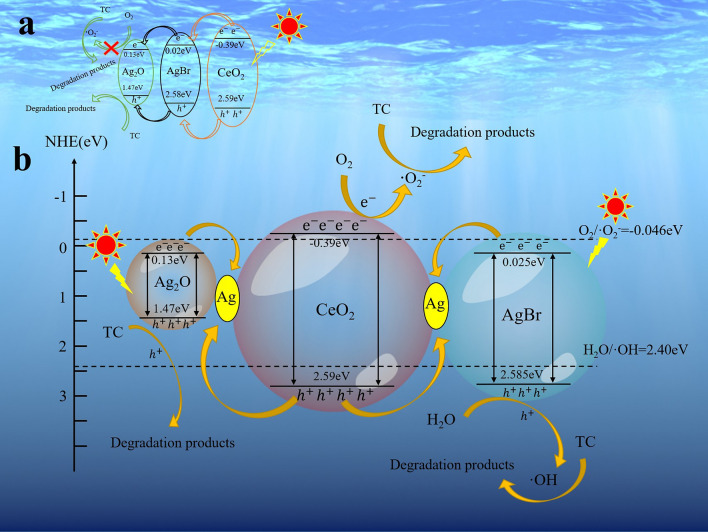
Schematic illustration of the proposed reaction mechanism for TC degradation under visible light irradiation based on the (**a**) conventional heterojunction; (**b**) double Z-scheme heterojunction.

**Scheme 1:**
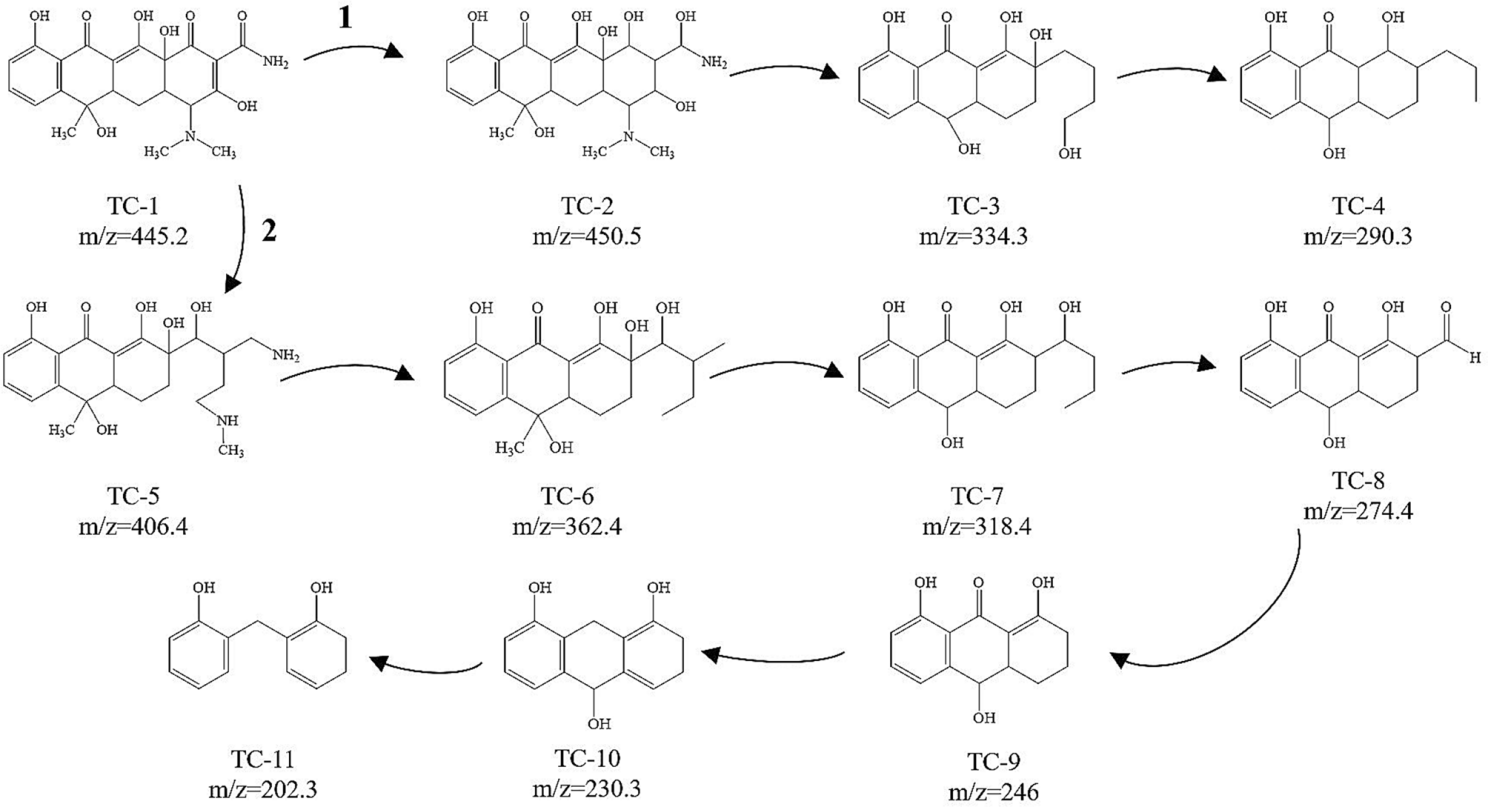
Schematic diagram of proposed TC photodegradation pathway by ACA-2.

**Scheme 2:**
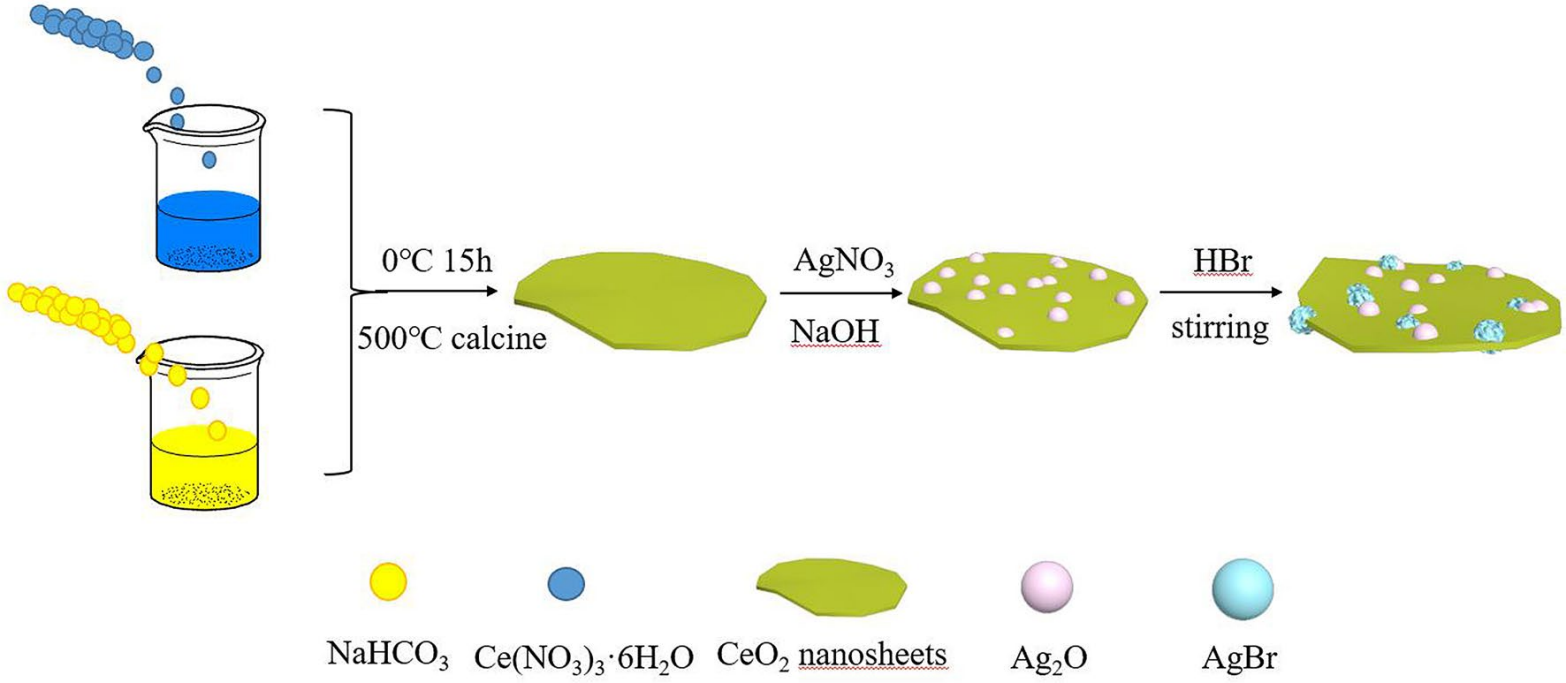
Schematic diagram of Ag_2_O/AgBr–CeO_2_ catalyst preparation process.

The mechanism of photodegradation of TC over the catalyst is shown in Fig. 8. According to the traditional type I heterojunction structure (Fig. 8a), CeO_2_ is excited to produce photoelectrons under visible light irradiation. The electrons on the CeO_2_ (CB) jump to the CB of AgBr and continue to transfer to silver oxide (CB), which follows the law of conservation of energy. Meanwhile, h^+^ on the surface of CeO_2_ (VB) should transfer to VB of AgBr. Holes don’t rest on AgBr (VB) and jump to the valence band of Ag_2_O. Holes could combine with H_2_O to form hydroxyl radicals (H_2_O/OH: 2.40 eV vs. NHE). However, e^−^ on the conduction band of Ag_2_O can’t combine with O_2_ to form **·**O_2_^−^ due to the higher conduction band of Ag_2_O (1.30 eV) than superoxide radical potential (O_2_/**·**O_2_^−^: − 0.046 eV vs. NHE)^39,47^. Therefore, photocatalyst doesn’t produce **·**O_2_^−^ to oxidize organic matter that is inconsistent with those of free radical capture experiments. Based on the above analysis, the conjecture of Scheme 1 is unreasonable, so the second mechanism is proposed to explain the reaction. According to Fig. 6b, it is clearly obvious that the XPS spectrum of used samples has the peak of Ag^0^. Thus, the Z-scheme heterojunction system with Ag NPs as the bridge between Ag_2_O, AgBr and CeO_2_ can be carried out under visible light. As presented in Fig. 8b, Ag_2_O and AgBr are excited to produce photogenic electrons (e^−^) and holes (h^+^) under visible light irradiation. In addition, the electrons in the conduction band of Ag_2_O are transferred to the Ag NPs working as the electronic medium. So in such a situation, an electron of Ag_2_O is attracted by the electron trap constructed by Ag NPs. In a similar way, e^-^ of AgBr (CB) also transfers to Ag NPs. In the meantime, the holes in the valence band of Ag_2_O are transferred to the Ag NPs that are combined with e^−^ of Ag NPs. Holes in the AgBr (VB) are in the same way. The electrons flowing into the Ag NPs rapidly recombine with the holes, leading to an accelerated charge transfer rate. Moreover, Ag NPs realize the spatial isolation of photoelectric pairs, which greatly limits the undesirable recombination. Electrons of CeO_2_ (CB) combine with oxygen in H_2_O to form superoxide radicals (O_2_/**·**O_2_^−^: − 0.046 eV vs. NHE) and **·**O_2_^−^ can transform TC to CO_2_ and H_2_O. Holes in the valence band of Ag_2_O (1.47 eV) reacts with TC to form CO_2_ and H_2_O. h^+^ in the AgBr (VB) reacts with H_2_O to form **·**OH because of the higher valence band of AgBr (2.58 eV) than H_2_O/**·**OH potential (H_2_O/**·**OH: 2.40 eV vs. NHE)^48^. And then hydroxyl radicals oxidize organic matter into degradation products. The Scheme 2 is consist of the free radical capture experiments of reactive species. In brief, the double Z-scheme heterojunction system with Ag_2_O/AgBr–CeO_2_ composites could point out a particular mechanism guess, which not only improves the efficiency of carrier separations but also consolidates its stability.

In order to further understand the degradation process of TC, the spectroscopy of the catalytic process of the intermediate products was detected by liquid chromatography-mass spectrometry^48^ (LC–MS, details are in the support material) (Scheme 1). Many intermediates were detected in the solution, and the corresponding mass spectra are shown in Supplementary Fig. S9. The product with a mass—charge ratio (*m/z*) of 445 is due to the TC molecule. In addition, the *m/z* values produced during the photocatalytic degradation of TC in the presence of ACA-2 were 450, 406, 362, 334, 318, 290, 274, 246, 230 and 202. Based on the previous reports and the analysis of the results, two possible degradation pathways were proposed. For the first route, the double bond on TC are reduced to TC-2 (*m/z* = 450). And then TC-2 (*m/z* = 450) deaminates to form TC-3 (*m/z* = 334). TC-4 (*m/z* = 290) was obtained by removing one of the hydroxyl groups from TC-3 (*m/z* = 334). The second pathway was from the TC (*m/z* = 445) to TC-5 (*m/z* = 406) via fracture of the double bond. Afterwards, TC-5 deaminates to form TC-6 (*m/z* = 362), and TC-6 removed methyl group to form TC-7 (*m/z* = 318). Hydroxide radical of TC-7 was changed into TC-8 (*m/z* = 274) after oxidation. As the same time, TC-8 removed a formyl group to form TC-9 (*m/z* = 246). TC-9 continued to break off a carbonyl group to get TC-10 (*m/z* = 230). Meanwhile, TC-10 break the ring and removed a hydroxymethyl to form TC-11 (*m/z* = 202). Finally, a part of intermediates can ultimately mineralized into H_2_O and CO_2_.

## Conclusions

All in all, a novel ternary Ag_2_O/AgBr–CeO_2_ photocatalyst was successfully prepared by the acid corrosion process of Ag_2_O/CeO_2_ that was gained through in situ loading of Ag_2_O onto CeO_2_ nanosheets. Thus, a double Z-scheme heterojunction degradation mechanism featuring Ag NPs as the bridge between the photogenerated electrons and holes was proposed, which can degrade 93.23% TC solution. Transient photocurrent response indexes that ternary Ag_2_O/AgBr–CeO_2_ composites demonstrate the ability to efficiently separate photo-generated electrons and holes. Free radical capture experiments indicate that the degradation of TC mainly relies on three factors (h^+^, O_2_^−^ and **·**OH). We believe that the novel three-way Ag_2_O/AgBr–CeO_2_ catalyst could have great potential in the field of energy and environmental protection. This work could provide a different Z-scheme heterojunction system to construct ternary catalysts improving the degradation efficiency.

## Material and methods

Cerium nitrate hexahydrate (Ce(NO_3_)_3_·6H_2_O), hydrobromic acid (HBr), silver nitrate (AgNO_3_), sodium hydroxide (NaOH) and Sodium Bicarbonate (NaHCO_3_) were all provided from Sino pharm chemical reagents Co., which were all analytical reagents. Rhodamine B and Tetracycline were provided from Alighting reagent Co.

### Synthesis of CeO_2_ microsheets

CeO_2_ microsheets were synthesized in a method of the low-temperature synthesis based on the previous report^49^. In details, 3 mmol Ce(NO_3_)_3_·6H_2_O was added to 30 ml H_2_O and stirred for 30 min at 0 ℃, which is solution A. Moreover, 0.75 g NaHCO_3_ also dissolved in 30 ml H_2_O, which is labeled as solution B. Solution B is dropwise added to solution A, the mixed solution stirred for 30 min. Then, the solution aged for 15 h. The powders were washed and dried at 60 °C for 12 h.

### Synthesis of Ag_2_O–CeO_2_

CeO_2_ microsheets (1 mmol, 30 ml) were dispersed in 40 ml of ultrapure water (solution A) with the ultrasound equipment, then 5.00 ml AgNO_3_ (0.1 M) was added slowly to solution A under vigorous stirring. The mixed solution stirred for 30 min at room temperature. Meanwhile, 5.00 ml NaOH (0.1 M) was added drop by drop to the above mixed solution with stirring for 2 h at dark environment. The products were washed five times with ultrapure water and ethyl alcohol, and collected after being dried at 60 °C for 12 h. The sample was marked as AOC.

### Synthesis of Ag_2_O/AgBr–CeO_2_

CeO_2_–Ag_2_O (2.30 g) samples were ultrasonically dispersed in 50 ml of distilled water. Then a certain volume of HBr (0.1 M) was added in the above solution with stirring for 4 h in a dark environment. Afterward, the products were washed five times by ultrapure water and dried at 60 °C for 12 h. The synthetic flowchart of Ag_2_O/AgBr–CeO_2_ is illustrated in Scheme 1. Based on the volume of HBr (1.00, 2.00, 3.00, 4.00, 5.00 ml), the products of 19.54 wt% Ag_2_O/7.92 wt% AgBr–CeO_2_, 14.23 wt% Ag_2_O/15.37 wt% AgBr–CeO_2_, 9.21 wt% Ag_2_O/22.39 wt% AgBr–CeO_2_, 4.48 wt% Ag_2_O/29.03 wt% AgBr–CeO_2_, 35.31 wt% AgBr–CeO_2_ were marked as ACA-1, ACA-2, ACA-3, ACA-4 and CAB.

### Characterization

It is convenient to analyze and study the crystal structure and material type of samples through X-Ray Diffraction (XRD, Rigaku Co., Japan, D/max 2500 VL/PC) and the field emission scanning electron microscopy (FESEM, JEOL-2100). Field Emission Transmission Electron Microscope (FETEM, JEM-2100F) achieved TEM and HRTEM photographs with an acceleration voltage of 200 kV. X-ray photoelectron spectroscopy (XPS, Thermos ESCALAB Xi+) performed an elemental analysis of the samples. UV–Vis diffuse reflectance spectra (DRS) were recorded on Cary 5000 using with BaSO_4_ as reference material. Electrochemical measurements were performed at Zahner electrochemical workstation using a standard three-electrode system. Detailed instructions are attached to the supporting information. Electron paramagnetic resonance (EPR) was used to verify the existence of **·**O_2_^−^ and **·**OH.

### Activity test of photocatalyst

The photocatalytic activity of composites was studied by the degradation of Rhodamine B (RhB) and tetracycline (TC) under the visible light irradiation (λ > 420 nm). The catalyst (0.025 g) was dispersed in the 50 ml solution with RhB or TC (the concentration of RhB or TC was 10 mg/l). The mixed solution stirred for 30 min in a dark environment to reach adsorption equilibrium. Afterward, the suspension was illuminated in visible light (500 w X lamp, Shanghai Qiqian Technology Co.). 4.00 ml suspensions that were achieved with every 15 min were centrifuged to remove catalysts. The concentration of the RhB (554 nm) and TC (357 nm) was received by the UV-1200 spectrophotometer at its maximum absorption wavelength.

## Supplementary information

Supplementary Information.
