# Supplementary material for: Photocatalytic degradation of organic dye and tetracycline by ternary Ag2O/AgBr–CeO2 photocatalyst under visible-light irradiation
Source: Sci Rep. 2021 Jan 8;11:85. doi: 10.1038/s41598-020-76997-0 (PMC7794347; doi:10.1038/s41598-020-76997-0)
Supplement: Supplementary file 1 — Supplementary Information. [file 41598_2020_76997_MOESM1_ESM.docx]

**Support information**

**Photocatalytic degradation of organic dye and tetracycline by** **ternary Ag_2_O/AgBr-CeO_2_ photocatalyst under visible-light irradiation**

Fu Su ^a^, Pengpeng Li ^b^, Jianshu Huang ^a^, Meijuan Gu ^a^, Zhiying Liu ^a*^, Yanhua Xu ^a,*^

^a^ Nanjing Tech University, Nanjing 211800, P. R. China

^b^ College of Chemistry and Materials Science, Nanjing Normal University, Nanjing 210023, P. R. China. These authors contributed equally: Fu Su and Pengpeng Li. Yanhua Xu, email: yanhuaxu18@hotmail.com.

**1. Experimental section**

*1.1 Electrochemical measurement*

The working electrodes were prepared by a drop-casting method on conductive glass (FTO) with an effective area of 1.0 × 1.0 cm^2^. In a typical process, 10 mg of the as-synthesized samples was added into 0.1 mL of ethyl alcohol, and the samples were sonicated for 0.5h to form a uniform slurry. The obtained slurry was spread onto the conductive surface of FTO to form a uniform film. Then, The working electrode was obtained by drying the chip at 80℃ for 6 h. The photocurrent was measured with an electrochemical station (Zahner) in a standard three-electrode system. An Ag/AgCl electrode was used as the reference electrode and a Pt foil as the counter electrode, while the above prepared films were working electrodes. Na_2_SO_4_ solution (0.2M) was used as the electrolyte. Mott−Schottky plots of the samples were carried on the same standard three-electrode at a frequency of 1 kHz. The measured potentials versus Ag/AgCl were converted to the normal hydrogen electrode (NHE) scale by *E*_NHE_ = *E*_Ag/AgCl_ + 0.197.

*1.2*. *Liquid chromatography-mass spectrometry*

The degradation intermediates of TC were identified by a LC-MS system (1290/6460 Triple Quad, Agilent) equipped with a Kromasil C18 column (250×4.6 mm, 5 µm). Eluants (flow rate: 0.2 mL/min): 0.1% (v/v) of formic acid aqueous solution (A) and acetonitrile (B). Injection volume: 2 µL. Column temperature: 30℃. Linear gradient elution process: initial 90% A was reduced to 10% A in 10 min and retained for 4 min. Then the mobile phase A returned to 90% in 1 min and maintained for 1 min. MS was conducted in the positive ion mode using an electrospray ionization (ESI) source under the following conditions: capillary voltage: 4.0 kV; gas (N_2_) flow rate: 11 L/min; gas temperature: 300℃; nebulization pressure: 15 psi.

**2. Results and discussions**

**
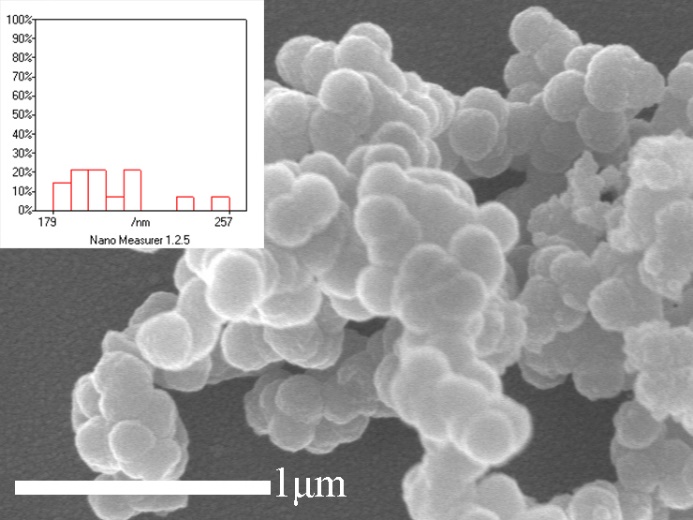
**

Fig. S1 SEM image of Ag_2_O


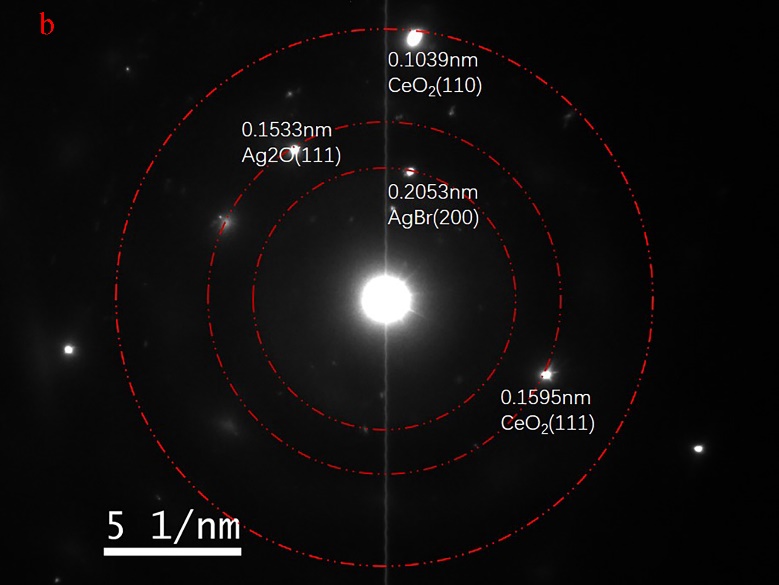


Fig. S2 SAED image of ACA-2

**
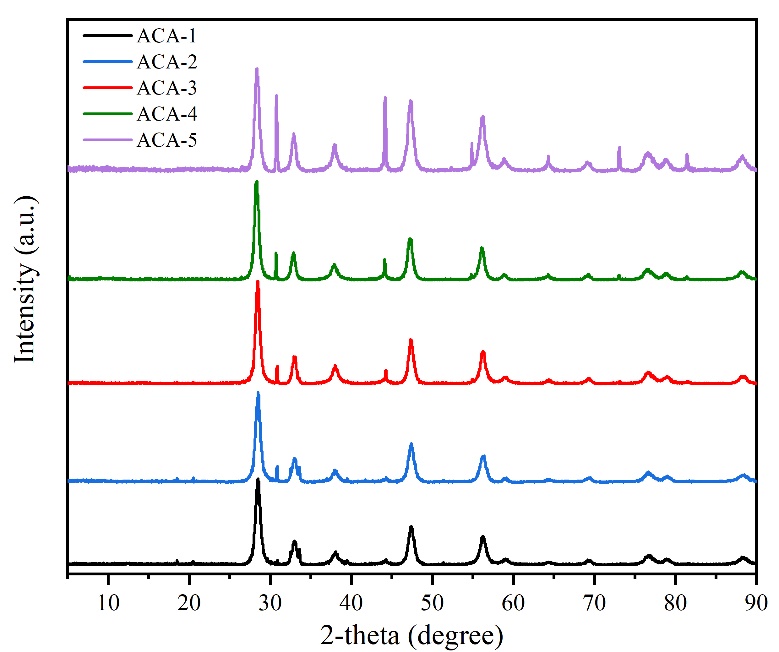
**
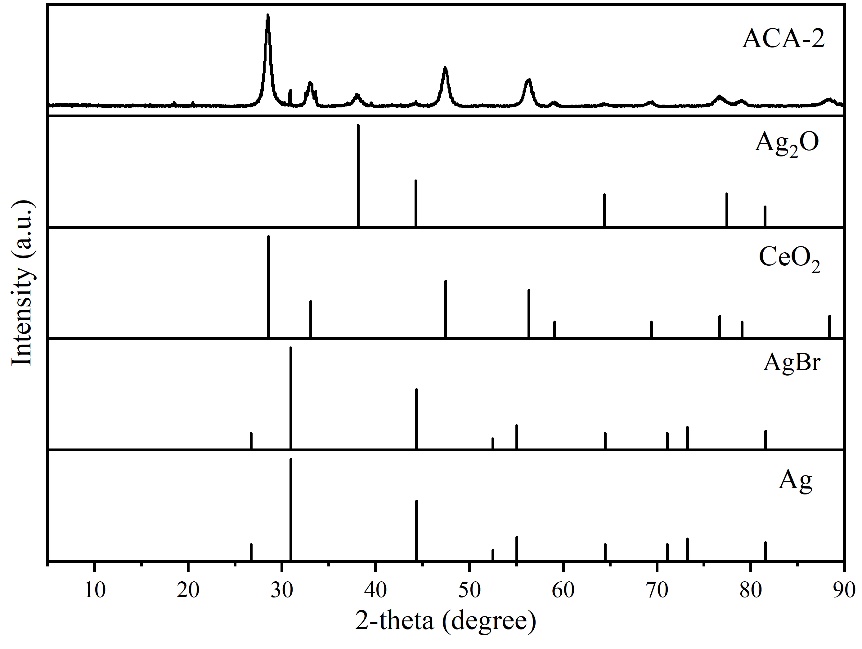


a

b

Fig. S3 (a) XRD spectra of ACA-X (X=1, 2, 3, 4) and CAB; (b) XRD standard comparison chart of Ag_2_O, AgBr, Ag and CeO_2_


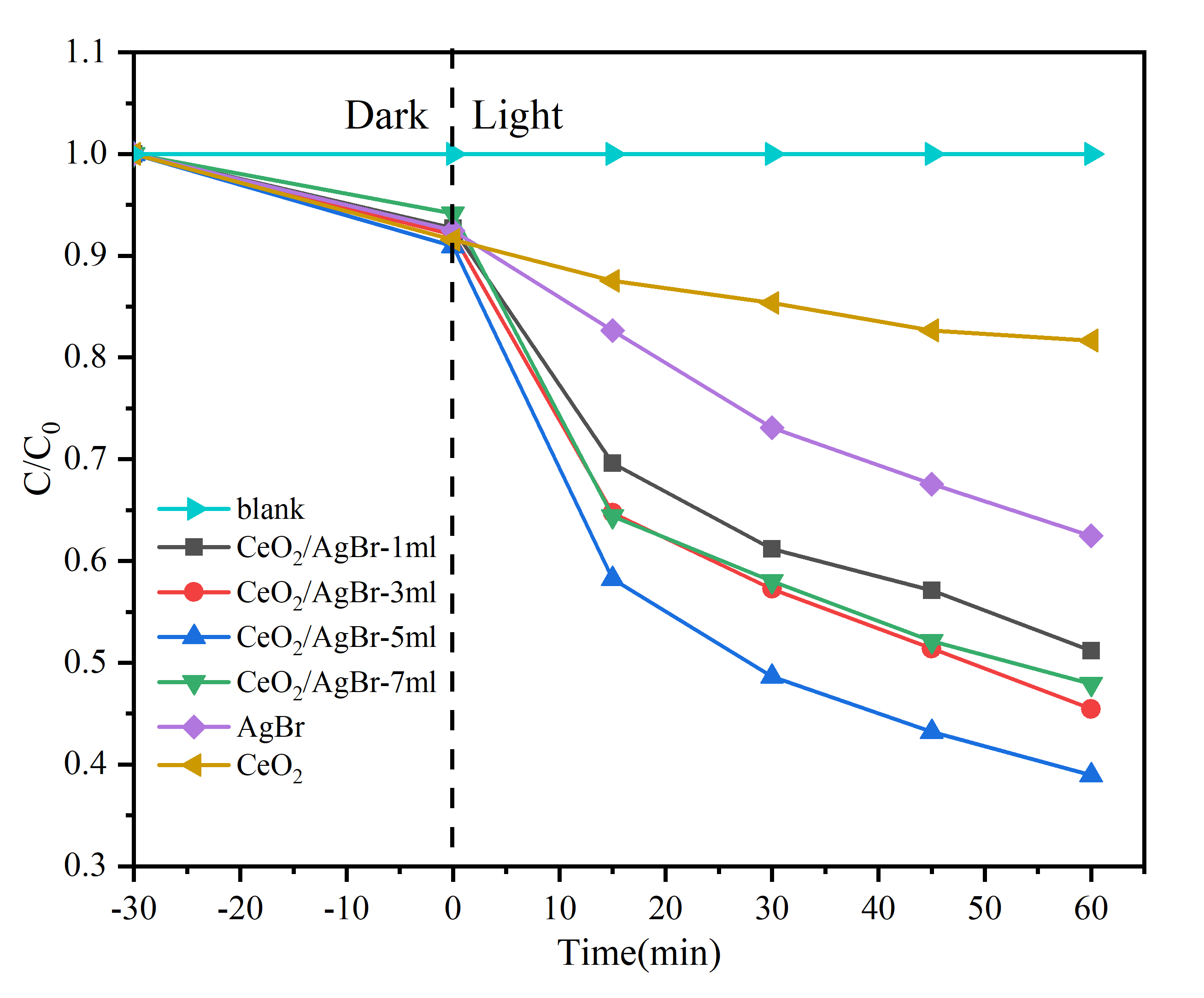


Fig. S4. Photocatalytic degradation RhB curves by AgBr/CeO_2_

b

a


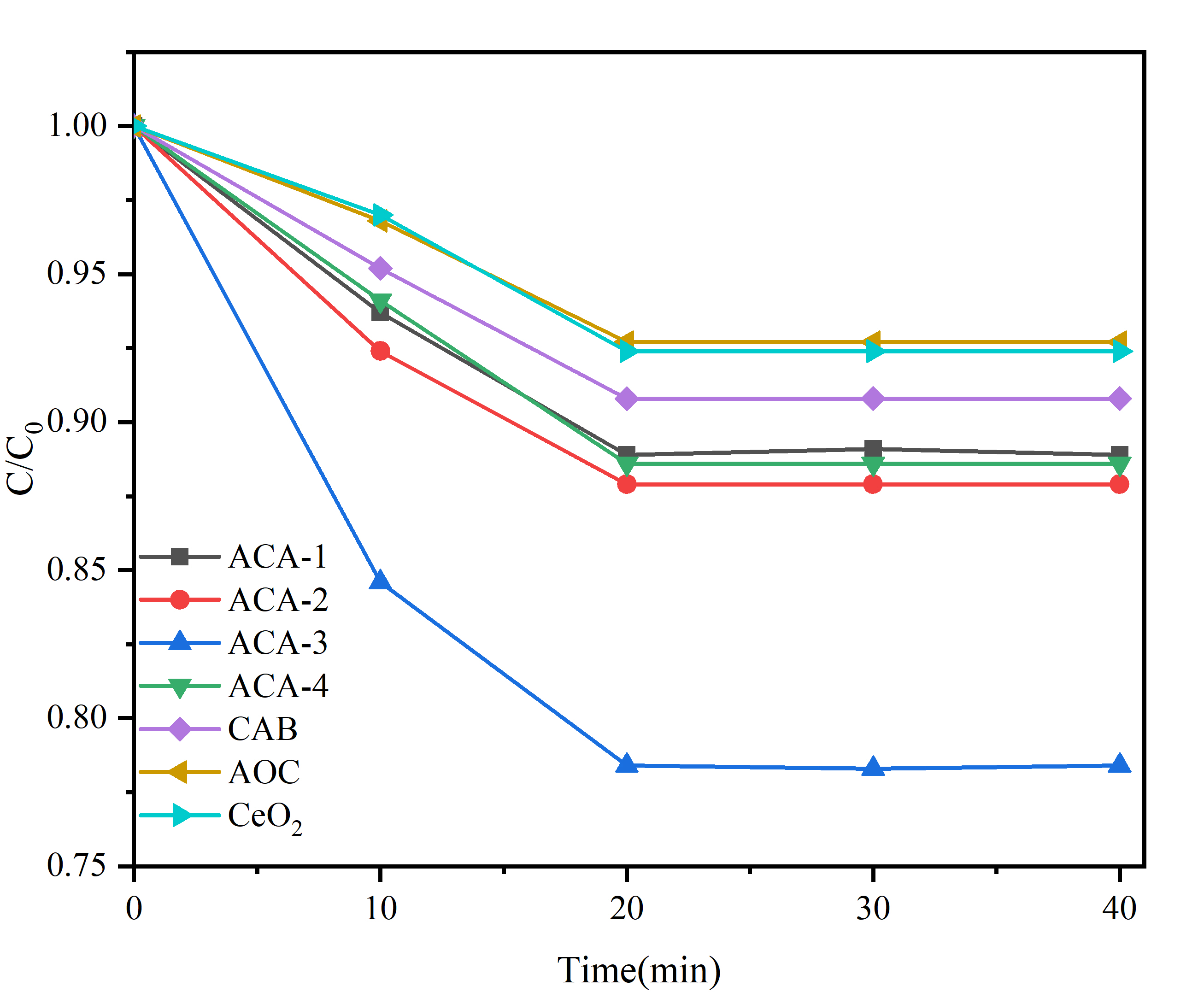

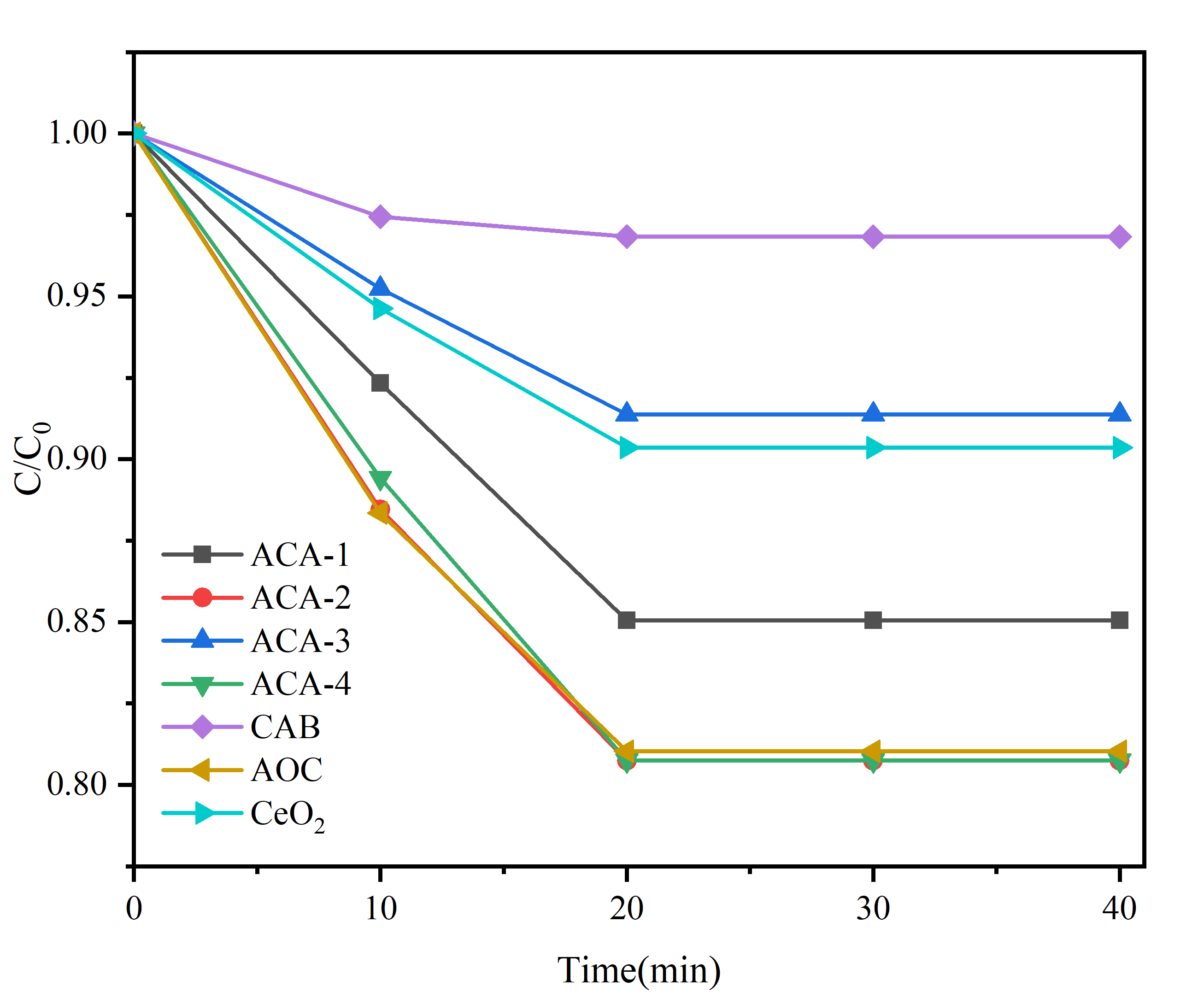


Fig. S5. Adsorption equilibrium of (a) RhB and (b) TC


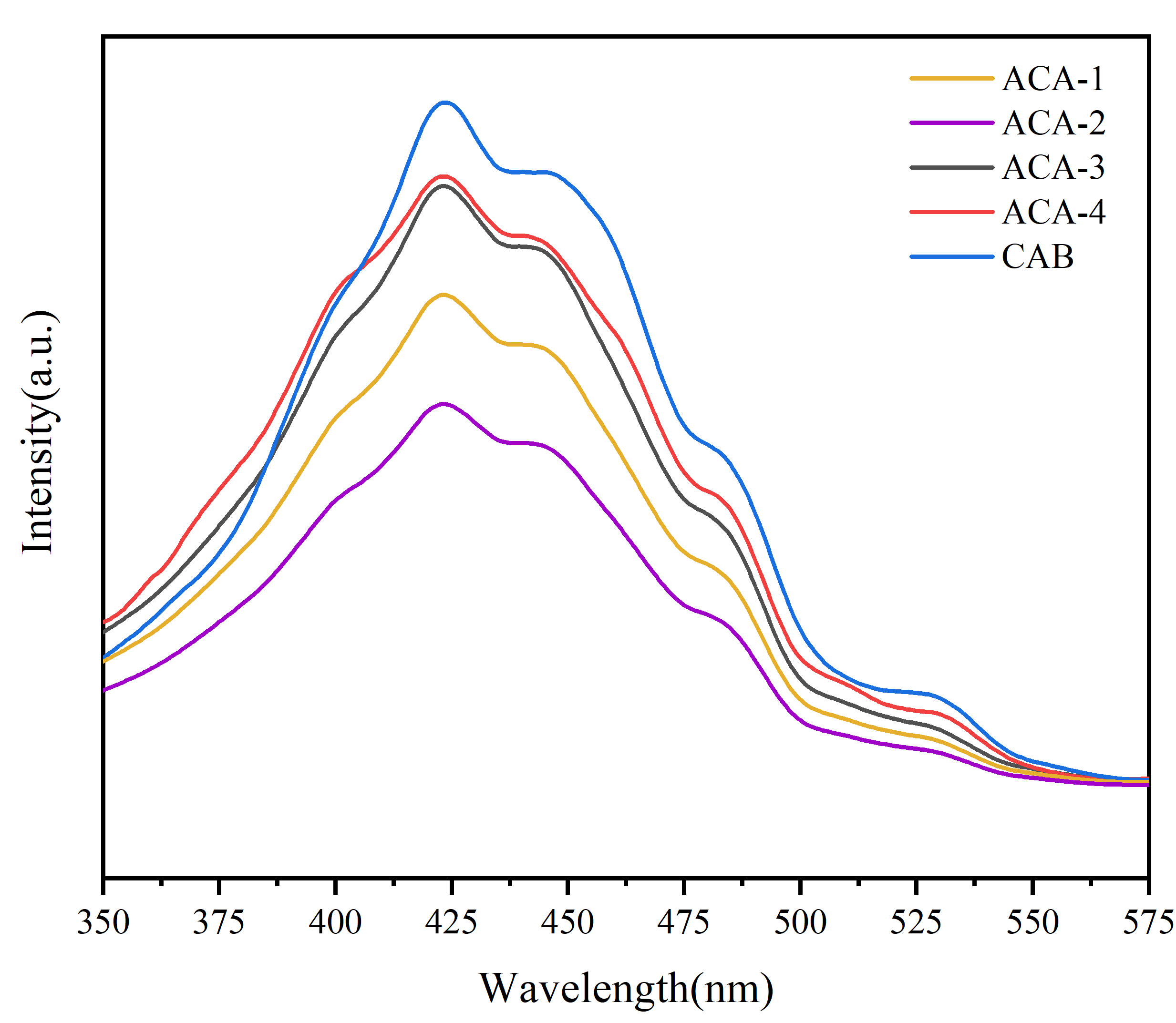

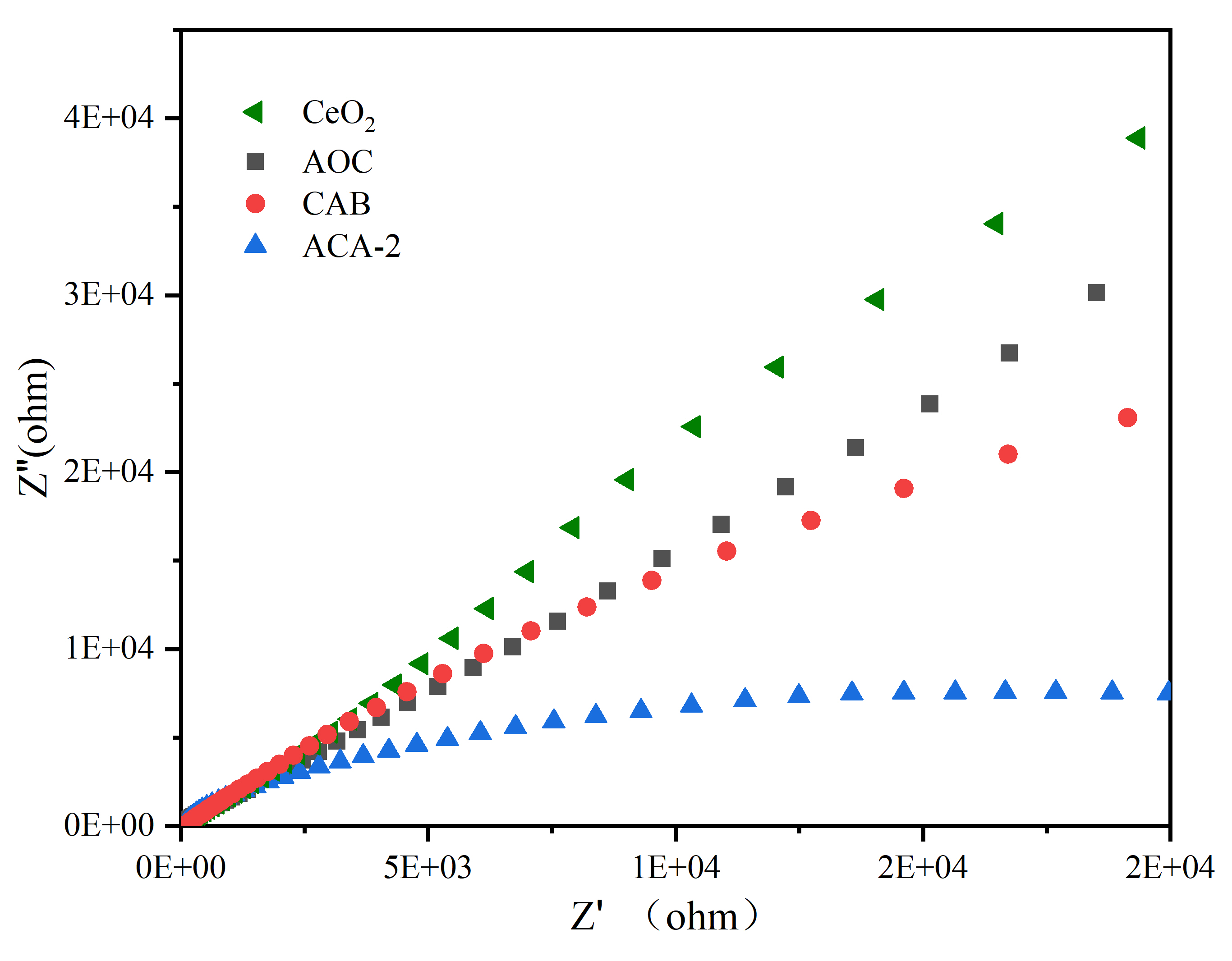


a

b

Fig. S6. (a) PL spectra of ACA-X (X=1, 2, 3, 4) and CAB; (b) EIS spectra of CeO_2_, AOC, CAB and ACA-2

b

a


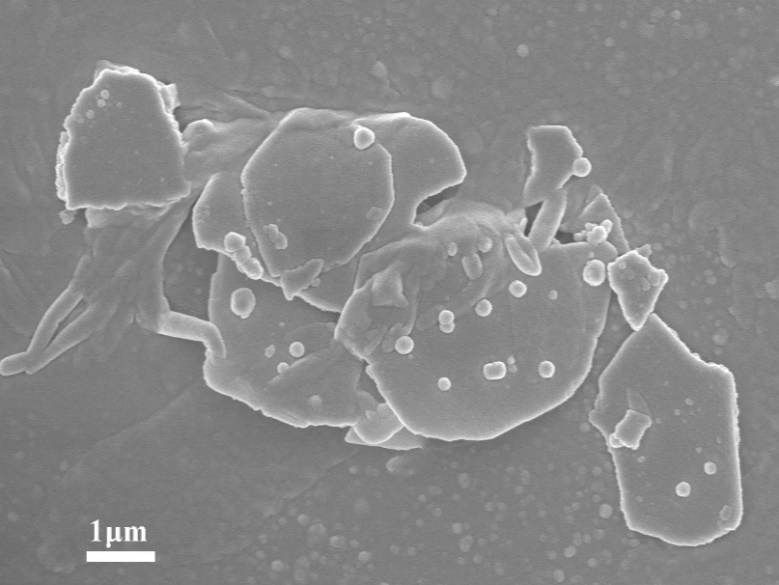

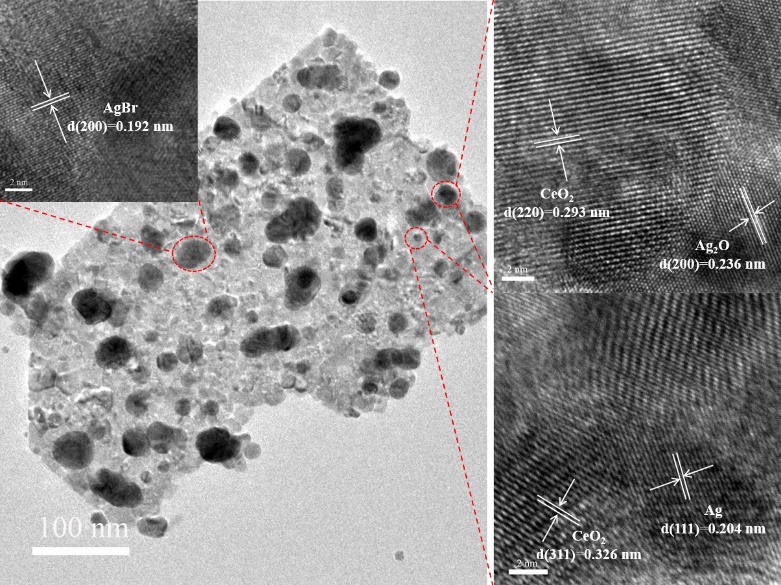


Fig.S7 (a) SEM image of used ACA-2; (b) TEM and HRTEM images of used ACA-2


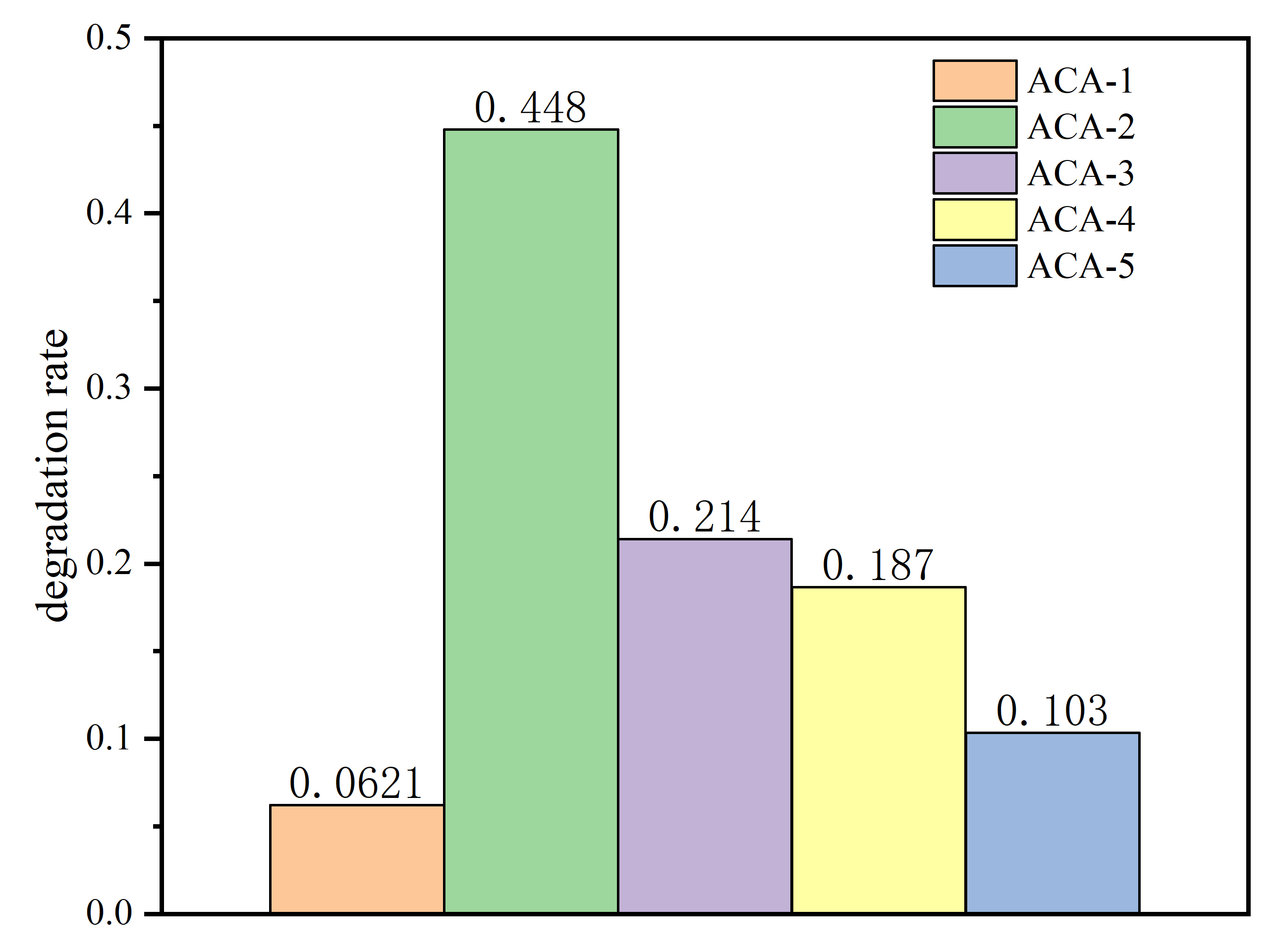


Fig. S8. TOC removal rate of ACA-X (X=1,2, 3, 4, 5)


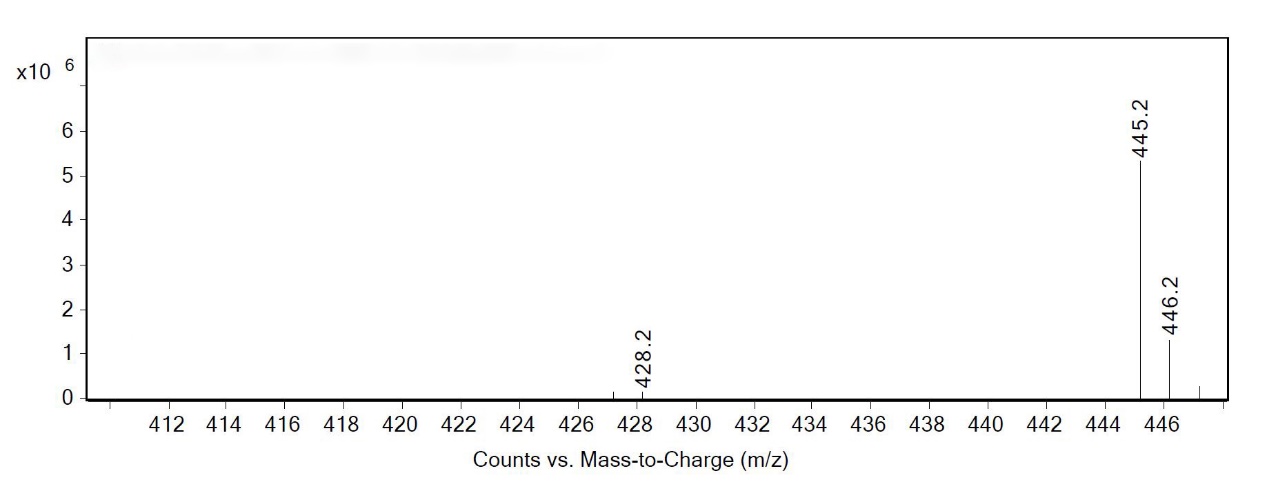


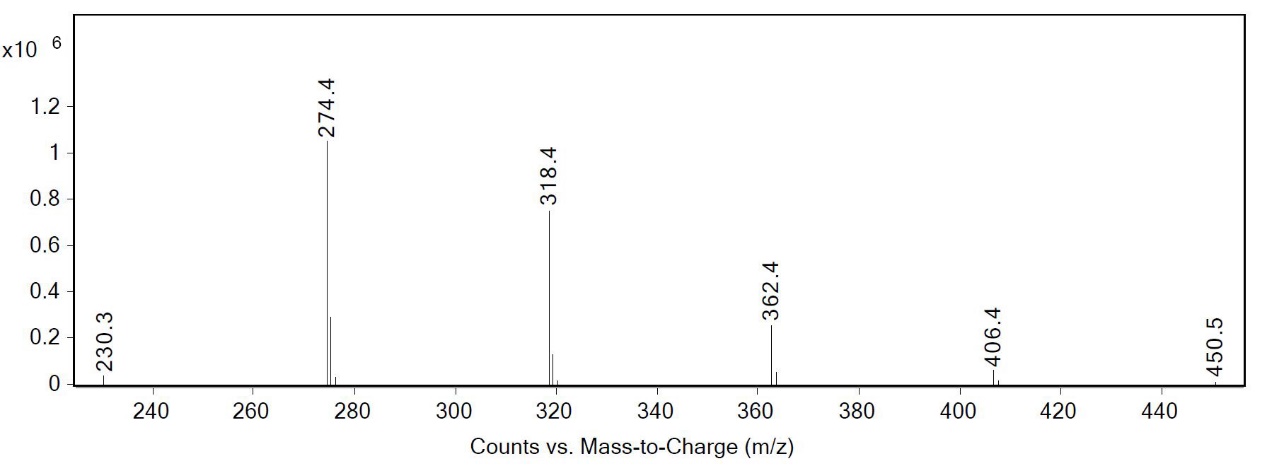

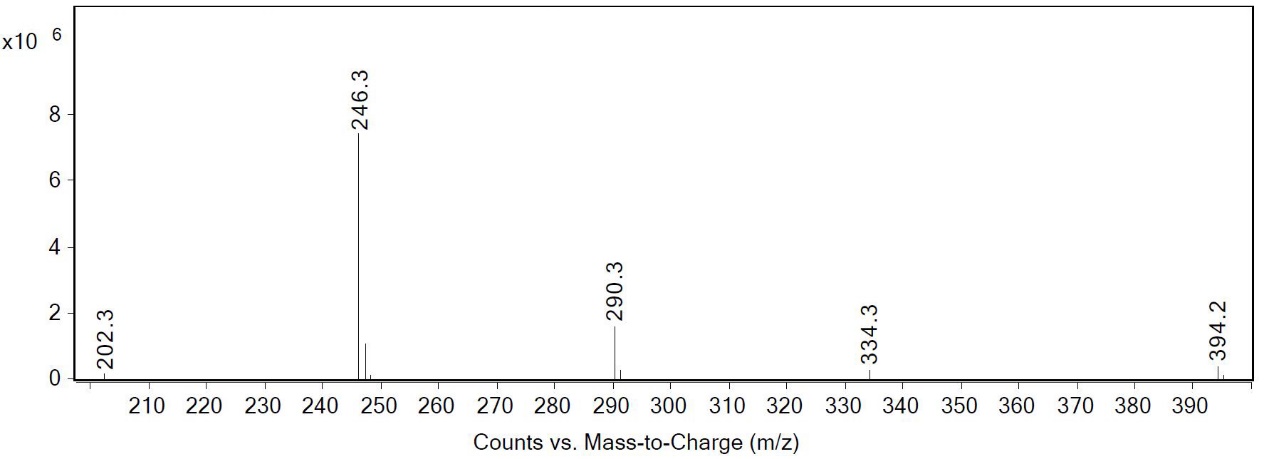
Fig. S9 MS spectra of possible intermediates of TC at different reaction time

**Table S1** Element content of ACA-2

| **Element Number** | **Element Symbol** | **Element Name** | **Atomic Conc.** | **Weight Conc.** |
| --- | --- | --- | --- | --- |
| 58 | Ce | Cerium | 18.31 | 54.84 |
| 8 | O | Oxygen | 72.29 | 24.73 |
| 47 | Ag | Silver | 7.30 | 16.84 |
| 35 | Br | Bromine | 2.11 | 3.60 |
